# Broadened assessments, health education and cognitive aids in the remote memory clinic

**DOI:** 10.3389/fpubh.2022.1033515

**Published:** 2022-12-07

**Authors:** Andrew P. Owens, Christine Krebs, Sajini Kuruppu, Anna-Katharine Brem, Tobias Kowatsch, Dag Aarsland, Stefan Klöppel

**Affiliations:** ^1^Department of Old Age Psychiatry, Institute of Psychiatry, Psychology and Neuroscience, King's College London, London, United Kingdom; ^2^University Hospital of Old Age Psychiatry and Psychotherapy, University of Bern, Bern, Switzerland; ^3^Institute for Implementation Science in Health Care, University of Zurich, Zurich, Switzerland; ^4^School of Medicine, University of St. Gallen, St. Gallen, Switzerland; ^5^Centre for Digital Health Interventions, Department Management, Technology, and Economics at ETH Zurich, Zurich, Switzerland

**Keywords:** online therapy, cognition, devices, dementia, serious games

## Abstract

The prevalence of dementia is increasing and poses a health challenge for individuals and society. Despite the desire to know their risks and the importance of initiating early therapeutic options, large parts of the population do not get access to memory clinic-based assessments. Remote memory clinics facilitate low-level access to cognitive assessments by eschewing the need for face-to-face meetings. At the same time, patients with detected impairment or increased risk can receive non-pharmacological treatment remotely. Sensor technology can evaluate the efficiency of this remote treatment and identify cognitive decline. With remote and (partly) automatized technology the process of cognitive decline can be monitored but more importantly also modified by guiding early interventions and a dementia preventative lifestyle. We highlight how sensor technology aids the expansion of assessments beyond cognition and to other domains, e.g., depression. We also illustrate applications for aiding remote treatment and describe how remote tools can facilitate health education which is the cornerstone for long-lasting lifestyle changes. Tools such as transcranial electric stimulation or sleep-based interventions have currently mostly been used in a face-to-face context but have the potential of remote deployment—a step already taken with memory training apps. Many of the presented methods are readily scalable and of low costs and there is a range of target populations, from the worried well to late-stage dementia.

## Introduction

The number of people aged 65 and older is predicted to almost double from ~900 million (12%) to 2 billion (22%) by 2050 (1), accompanied by substantial increases in the prevalence of dementia (2). Although many cognitive functions remain high until well into the sixth decade, age is associated with cognitive and functional decline and is the most important risk factor for dementia. There is strong consensus among experts that the neuropathological processes leading to dementia take decades (3). Importantly, early accurate etiologic diagnosis is also in line with public attitudes. Many people want to know if they have or may develop Alzheimer's disease (AD) so that targeted treatment can be provided as early as possible. While risk assessment typically addresses individuals free of cognitive symptoms, early diagnosis refers to a timely diagnostic work-up when symptoms fulfilling diagnostic criteria of dementia or mild cognitive impairment (MCI) are present.

One metric of dementia diagnosis is the presence of cognitive symptoms impairing daily life. This can formally be measured by defined activities of daily living (ADLs), making them an ideal target for sensor-based assessment. MCI is indicative of risk for future cognitive decline, with up to 15% of those diagnosed with MCI developing dementia yearly (4), most frequently due to AD. Originally, MCI was considered not to affect ADL, but subsequent studies (5, 6) and metanalyses report instrumental ADLs are already impaired in MCI (7, 8).

The COVID-19 pandemic has heightened the need for remote (i.e., virtual) assessments. Advances in healthcare technology, including electronic health records, healthcare platforms and wireless communications, have made the remote collection of clinically relevant data possible. In this context, Remote Measurement Technologies (RMTs), refers to, “any mobile technology that enables monitoring of a person's health status through a remote interface, with the data then either transmitted to a health care provider for review or to be used as a means of education for the users themselves” (9). RMTs may include a variety of sensors that detect changes in health status, offering a unique opportunity to accurately and continuously track and measure changes. RMTs can objectively, actively, and passively collect numerous data points during every-day routines.

The current paper extends related publications (10, 11) by broadening the range of assessments and target groups and by describing treatment options currently being transferred from face-to-face to remote settings. We also highlight the need to assess psychiatric conditions, particularly depressive symptoms to correctly interpret identified cognitive impairment, tailor therapeutic interventions and improve outcome.

## Assessment

### Remote assessment of ADL

A multitude of tools exists to assess cognition, from computer-based online platforms to mobile (typically smartphone-based) solutions and have been reviewed recently (10). We focus on ADL detection. Functional status, as measured by ADLs, also has prognostic purposes, as those with MCI and mild functional impairments at baseline are more likely to convert to overt dementia. Functional status is also increasingly recognized as a relevant outcome in treatment trials and current treatments. From the point of sensor technology, their strength is to measure ADL directly (12) and in real-world situations. Current measures of ADL functioning are intermittent and subjective, as they are based on a retrospective account of patients and/or relatives. Actual demonstrations of ADL in the clinic are rare, although virtual reality may play a facilitating role in the future (13). Importantly, in current clinical practice, they are neither evaluated in the real-life context of the patient nor measured continuously. As with assessments of cognition, ADL are influenced by somatic and psychiatric comorbidities, which should be evaluated simultaneously.

### Broadening the scope of assessments

Cognitive symptoms do not always indicate symptoms of neurodegenerative processes. In addition to other somatic conditions (e.g., stroke, systemic diseases, etc.), depressive symptoms are typically associated with objective cognitive decline (14), and both are frequent in the elderly.

Around 20% of adults aged 60 years and over are afflicted with psychiatric disorders, with dementia (5%) and depression (7%) being the most common and debilitating (https://www.who.int/news-room/fact-sheets/detail/mental-health-of-older-adults). The COVID-19 pandemic has further highlighted the mental health vulnerability of the elderly, including cognitive decline, depression, anxiety and loneliness (15). These psychiatric symptoms are typically measured using self-ratings or clinical assessments. They can, however, also be detected from voice (16, 17). Changes in movements or locations measured using GPS-data as well as changes in the pattern of smartphone use might further indicators of depressive symptoms (18).

**Figure 1:**
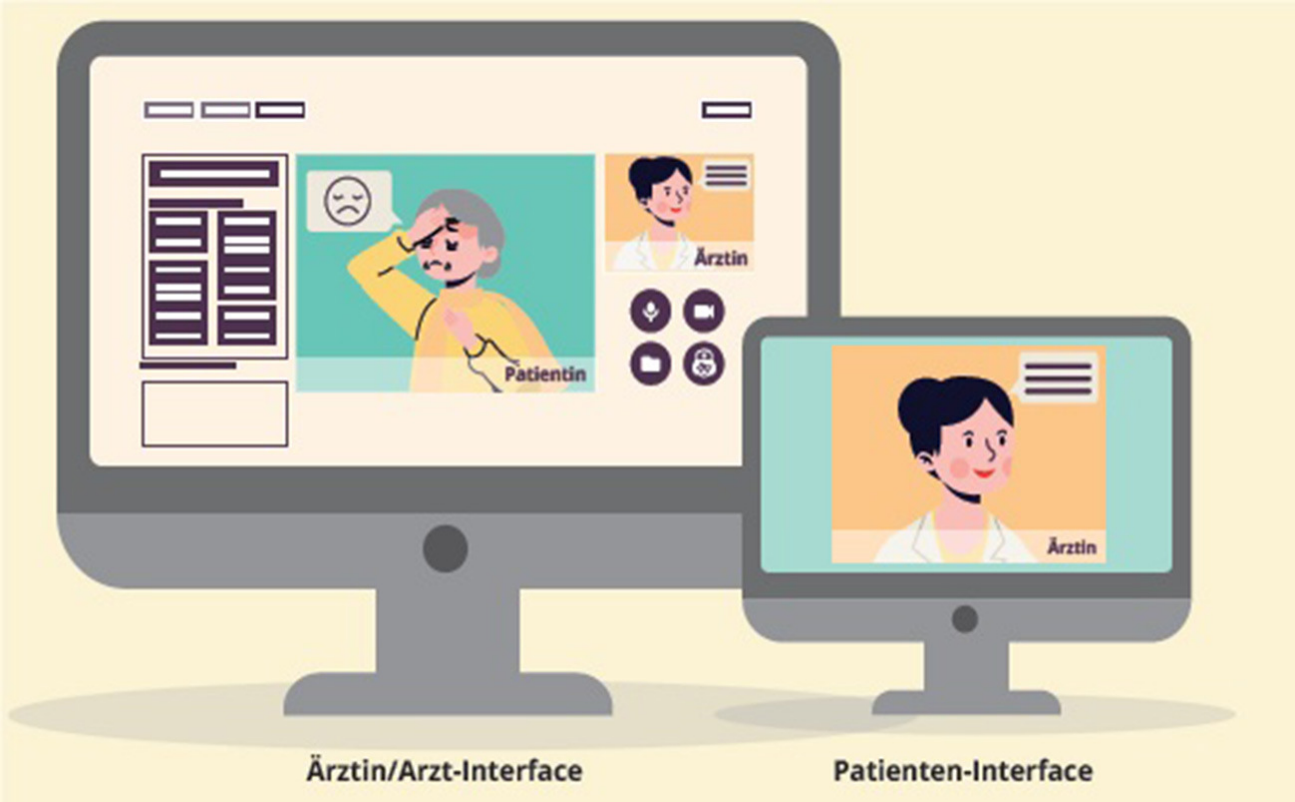
Interface for the remote cognitive testing. The patient's screen will typically show examiner (= “Ärztin”) or test material (e.g., objects in a naming task). The examiner sees the patient (= “Patientin”), a duplication of the patient's screen (upper right) and tool lists to conduct and record the testing.

It is noteworthy that these assessments can be extended to more severely impaired patients who are unable to use a computer or other digital device independently. Solutions currently under investigation include the installation of computers for cognitive assessments in the practice of GPs or local pharmacies. In those settings, the assessment is guided by a clinician, and patients' responses are monitored by audio and video, negating the use of keyboard or mouse (Figure 1). The concept has recently been extended by installing the assessment computer in a van to reach patients living in remote areas (19).

### Broadening the target population

In care homes, 70% of residents have dementia or severe memory impairment (20) and 98% of those living with dementia exhibit neuropsychiatric symptoms, such as anxiety, agitation and depression, which again, have been exacerbated by the COVID-19 pandemic and related social distancing measures put in place to minimize infection risk in vulnerable populations (21). Older studies indicate low detection rates for depression and cognitive impairment in care homes due to a lack of valid and reliable assessment measures (22), which remote assessment must address (23).

Where those with severe dementia form one end of the spectrum, older people without cognitive problems or related concerns may form the other. They increasingly like to contribute to science and care as part of the growing trend of “Citizen Science”. While this produces relevant scientific findings, it also empowers participants by giving them an active and informed role in their own healthcare. For example, Join Dementia Research (https://www.joindementiaresearch.nihr.ac.uk/) is a platform where interested volunteers can register to take part in dementia research. Another case in point is the PROTECT study (www.protectstudy.org.uk). This is an online longitudinal study of a healthy aging (>50 years) population funded by the National Institute of Health and Research (NIHR) for 25 years with a recruitment target of 50,000 participants. In a recent study in which gamified brain training tasks were deployed *via* the PROTECT platform, after 6 months, brain training was associated with significant improvements in ADL scores in end-users aged >60, and significant improvements in reasoning and verbal learning occurred in end-users aged >50 years comparative to those who didn't play the reasoning and problem-solving games. The need for standard clinical practice to be supplemented by participant-led lifestyle behaviors was emphasized by the finding that the brain training games had to be played five times per week to evidence their positive effects. Improved and more frequent diagnostics through RMT allow for better tailored interventions and higher training frequency. Currently, there are not many treatment options available in care homes.

## Remote interventions and monitoring

While RMT focuses on measuring cognitive function, it is often closely associated with a training aspect. This is obvious for cognitive assessments when implemented as brain training or serious games, as in the PROTECT study. Here, the gaming element can be the obvious characteristic from the end user's perspective. However, in-game performance could allow deducting cognitive performance in well-established categories such as executive functioning or core sub-functions such as working memory. While these domains are required for most classic computer games, specifically designed games also allow testing and training domains such as episodic memory, naming or spatial rotation which are typically affected by AD. Among the strengths of the PROTECT study are the number of participants and the long duration. A study on serious games for cognitive training currently run in Switzerland is specifically geared toward domains affected by AD (24). The study also seeks to investigate the neuronal correlates of the intervention using functional brain imaging. The aim is to provide insights into the adaption of cognitive networks (working memory, attention span, and episodic memory) as a result of gamified training. These findings could then be the basis for designing more efficient training protocols or providing protocols more geared to the individual.

Challenges for routine implementation of serious game-based training remain low adherence, particularly when elderly participants ought to be playing alone, and the lack of data on long-term benefits, such as functioning in daily life. While serious game-based cognitive training is now a well-established online and remote intervention, its effects may be augmented through non-invasive brain stimulation.

Transcranial electric stimulation (tES) is an example of a remotely applicable device purely geared toward treatment. TES is an umbrella term for methods that apply tiny currents to the brain through skin electrodes. The currents are too low to elicit action potentials but may alter synaptic potentials. For these methods to affect performance, they need to be applied either before or during a specific task. Studies have shown efficacy across a wide range of cognitive, affective, and motor domains (e.g., dexterity, language learning, and memory), but others have challenged the robustness and longevity of these findings. A recent study comparing different protocols of tES (transcranial direct current stimulation against alternating currents or placebo) combined with cognitive training found no additional benefits from tES in elderly healthy participants. Instead, direct current stimulation provided additional benefits for participants performing particularly low at baseline (25). A recent review highlights the need for further investigation of long-term and transfer effects of combined approaches in MCI and dementia (26). TES devices are commercially available and are currently investigated for home use (27, 28).

Notably, remote cognitive training can be combined with tES and other interventions such as physical exercise, dietary measures etc. Multi-domain interventions appear to result in improved cognitive outcomes in populations at risk of cognitive decline [e.g., (29)]. Such multi-domain approaches could be integrated into flexible internet-based platforms from which individualized training plans could be compiled based on individual preferences and needs.

Many studies have highlighted the link between sleep quality and dementia and encouraging basic measures of sleep hygiene are warranted. Of recent interest are methods using closed-loop acoustic stimulation to improve memory consolidation (30). These methods typically detect slow-waves in deep sleep and augment their duration through acoustic entrainment by playing pink noise signals without disrupting the sleep. Similar to tES, more and more devices are currently being developed for home use (dreem.com/science; sleeploop.ch/de/home).

## Outlook

The pandemic has accelerated the spread of RMTs, and it is clear that they will play an even more important role in diagnostics and treatments in the future. Virtual reality is already part of the gaming industry and will soon become the standard for assessing fitness to drive (31). Voice features will also gain importance as studies indicate their ability to detect cognitive impairment (32–34).

In the future, we expect a much wider distribution of just-in-time lifestyle interventions (35, 36). They could detect current situations (e.g., GPS to detect somebody approaching an elevator or restaurant) and recommend dementia-preventing behavior (i.e., taking the stairs or eating a healthy diet). Artificial intelligence could learn the most suitable situations and methods of prompting and focus on those. Similarly, the individual pattern of health related behavior could inform oneself about biggest challenges (when the unhealthy behavior prevailed) or where frequently shown beneficial behavior could be further encouraged simply be making it transparent how often desirable behavior is already adopted.

Relatively little attention in the context of RMT went into its potential to improve health literacy. Although many online resources exist, few benefit from the potential of online methods by combining video with text and quizzes (https://www.alzu.org). Health literacy is necessary for oneself to take over responsibility for health behavior and is increasingly requested also be seniors and in the medical context. It is likely that a mixture between individual (remote) counseling and access to Internet resources would provide the best results. The same is likely for knowledge transfer as well as remote treatment options where adherence benefits from interactions with other humans. This would mirror the status in the field of app-supported psychotherapy where blended approaches typically outperform app-only approaches (37–44). In other words, remote approaches are unlikely to replace personal interaction with therapists but offer the opportunity to increase training frequency, precision interventions and monitoring of progress.

Lastly, user-friendliness of the technical solutions requires special attention in cognitively impaired or depressed individuals. Fortunately, the technical infrastructure and skills necessary to build health interventions are now easily available (e.g., www.mobile-coach.eu).

At least when the first cohorts of digital natives age, skills and experience with a broad range of devices are also given. However, eyesight and dexterity will still pose challenges in using devices with a small form factor (e.g., smartphones or smartwatches). Involving seniors in designing the future of RMTs and tailored interventions remains key.

## Author contributions

AO and SKl writing—original draft preparation. CK, SKu, A-KB, TK, and DA writing—review and editing. All authors have read and agreed to the published version of the manuscript.

## References

[1] United Nations Department Department of Economic and Social Affairs Population Division. World Population Prospects 2019: Highlights. ST/ESA/SER.A/423. (2019).

[2] GBD 2019 Dementia Forecasting Collaborators. Estimation of the global prevalence of dementia in 2019 and forecasted prevalence in 2050: an analysis for the Global Burden of Disease Study 2019. Lancet Public Health. (2022) 7:e105–25. 10.1016/S2468-2667(21)00249-8 34998485 PMC8810394

[3] Livingston G Huntley J Sommerlad A Ames D Ballard C Banerjee S. Dementia prevention, intervention, and care: 2020 report of the Lancet Commission. Lancet. (2020) 396:413–46. 10.1016/S0140-6736(20)30367-6 32738937 PMC7392084

[4] Dunne RA Aarsland D O'Brien JT Ballard C Banerjee S Fox NC. Mild cognitive impairment: the Manchester consensus. Age Ageing. (2021) 50:72–80. 10.1093/ageing/afaa228 33197937 PMC7793599

[5] Pérès K Chrysostome V Fabrigoule C Orgogozo JM Dartigues JF Barberger-Gateau P. Restriction in complex activities of daily living in MCI: impact on outcome. Neurology. (2006) 67:461–6. 10.1212/01.wnl.0000228228.70065.f1 16894108

[6] Lee MT Jang Y Chang WY. How do impairments in cognitive functions affect activities of daily living functions in older adults? PLoS ONE. (2019) 14:e0218112. 10.1371/journal.pone.0218112 31173607 PMC6555549

[7] Perneczky R Pohl C Sorg C Hartmann J Tosic N Grimmer T. Impairment of activities of daily living requiring memory or complex reasoning as part of the MCI syndrome. Int J Geriat Psychiatry. (2006) 21:158–62. 10.1002/gps.1444 16416470

[8] Jekel K Damian M Wattmo C Hausner L Bullock R Connelly PJ. Mild cognitive impairment and deficits in instrumental activities of daily living: a systematic review. Alzheimer's Res Therapy. (2015) 7:1–20. 10.1186/s13195-015-0099-0 25815063 PMC4374414

[9] Davis MM Freeman M Kaye J Vuckovic N Buckley DI. A systematic review of clinician and staff views on the acceptability of incorporating remote monitoring technology into primary care. Telemed e-Health. (2014) 20:428–38. 10.1089/tmj.2013.0166 24731239 PMC4011427

[10] Owens AP Ballard C Beigi M Kalafatis C Brooker H Lavelle G. Implementing remote memory clinics to enhance clinical care during and after COVID-19. Front Psychiatry. (2020) 11:579934. 10.3389/fpsyt.2020.579934 33061927 PMC7530252

[11] Owens AP Hinds C Manyakov NV Stavropoulos TG Lavelle G Gove D. Selecting remote measurement technologies to optimize assessment of function in early Alzheimer's disease: a case study. Front Psychiatry. (2020) 11:582207. 10.3389/fpsyt.2020.582207 33250792 PMC7674649

[12] Urwyler P Stucki R Rampa L Müri R Mosimann UP Nef T. Cognitive impairment categorized in community-dwelling older adults with and without dementia using in-home sensors that recognise activities of daily living. Sci Rep. (2017) 7:42084. 10.1038/srep42084 28176828 PMC5296716

[13] Vallejo V Wyss P Rampa L Mitache AV Müri RM Mosimann UP. Evaluation of a novel serious game based assessment tool for patients with Alzheimer's disease. PLoS ONE. (2017) 12:e0175999. 10.1371/journal.pone.0175999 28472049 PMC5417424

[14] Leyhe T Reynolds CF Melcher T Linnemann C Kloppel S Blennow K. A common challenge in older adults: classification, overlap, and therapy of depression and dementia. Alzheimer's Dement J Alzheimer's Assoc. (2017) 13:59–71. 10.1016/j.jalz.2016.08.007 27693188

[15] Vik-Mo AO Giil LM Borda MG Ballard C Aarsland D. The individual course of neuropsychiatric symptoms in people with Alzheimer's and Lewy body dementia: 12-year longitudinal cohort study. Br J Psychiatry. (2020) 216:43–8. 10.1192/bjp.2019.195 31506117

[16] Cho G Yim J Choi Y Ko J Lee S-H. Review of machine learning algorithms for diagnosing mental illness. Psychiatry Invest. (2019) 16:262–9. 10.30773/pi.2018.12.21.2 30947496 PMC6504772

[17] Low DM Bentley KH Ghosh SS. Automated assessment of psychiatric disorders using speech: a systematic review. Laryngosc Invest Otolaryngol. (2020) 5:96–116. 10.1002/lio2.354 32128436 PMC7042657

[18] Moshe I Terhorst Y Opoku Asare K Sander LB Ferreira D Baumeister H. Predicting symptoms of depression and anxiety using smartphone and wearable data. Front Psychiatry. (2021) 12:625247. 10.3389/fpsyt.2021.625247 33584388 PMC7876288

[19] Zeghari R Guerchouche R Tran Duc M Bremond F Lemoine MP Bultingaire V. Pilot study to assess the feasibility of a mobile unit for remote cognitive screening of isolated elderly in rural areas. Int J Environ Res Public Health. (2021) 18:6108. 10.3390/ijerph18116108 34198917 PMC8201036

[20] Alzheimer's Society,. Facts for the Media About Dementia. Alzheimer's Society (2022). Available online at: https://www.alzheimers.org.uk/about-us/news-and-media/facts-media (accessed November 14, 2022).

[21] Velayudhan L Aarsland D Ballard C. Mental health of people living with dementia in care homes during COVID-19 pandemic. Int Psychogeriat. (2020) 32:1253–4. 10.1017/S1041610220001088 32487278 PMC7302947

[22] Worden A Challis DJ Pedersen I. The assessment of older people's needs in care homes. Aging Mental Health. (2006) 10:549–57. 10.1080/13607860600637794 16938690

[23] Saner H Schütz N Botros A Urwyler P Buluschek P du Pasquier G. Potential of ambient sensor systems for early detection of health problems in older adults. Front Cardiovasc Med. (2020) 7:110. 10.3389/fcvm.2020.00110 32760739 PMC7373719

[24] Brill E Krebs C Falkner M Peter J Henke K Züst M. Can a serious game-based cognitive training attenuate cognitive decline related to Alzheimer's disease? Protocol for a randomized controlled trial. BMC Psychiatry. (2022) 22:552. 10.1186/s12888-022-04131-7 35962371 PMC9373273

[25] Krebs C Peter J Wyss P Brem A-K Klöppel S. Transcranial electrical stimulation improves cognitive training effects in healthy elderly adults with low cognitive performance. Clin Neurophysiol. (2021) 132:1254–63. 10.1016/j.clinph.2021.01.034 33875372

[26] Cruz Gonzalez P Fong KNK Chung RCK Ting K-H Law LLF Brown T. Can transcranial direct-current stimulation alone or combined with cognitive training be used as a clinical intervention to improve cognitive functioning in persons with mild cognitive impairment and dementia? A systematic review and meta-analysis. Front Hum Neurosci. (2018) 12:416. 10.3389/fnhum.2018.00416 30386223 PMC6198143

[27] Charvet LE Shaw MT Bikson M Woods AJ Knotkova H. Supervised transcranial direct current stimulation (tDCS) at home: a guide for clinical research and practice. Brain Stimul. (2020) 13:686–93. 10.1016/j.brs.2020.02.011 32289698

[28] Palm U Kumpf U Behler N Wulf L Kirsch B Wörsching J. Home use, remotely supervised, and remotely controlled transcranial direct current stimulation: a systematic review of the available evidence. Neuromod Technol Neural Interface. (2018) 21:323–33. 10.1111/ner.12686 28913915

[29] Ngandu T Lehtisalo J Solomon A Levälahti E Ahtiluoto S Antikainen R. A 2 year multi-domain intervention of diet, exercise, cognitive training, and vascular risk monitoring vs. control to prevent cognitive decline in at-risk elderly people (FINGER): a randomised controlled trial. Lancet. (2015) 385:2255–63. 10.1016/S0140-6736(15)60461-5 25771249

[30] Wunderlin M Züst MA Fehér KD Klöppel S Nissen C. The role of slow wave sleep in the development of dementia and its potential for preventative interventions. Psychiatry Res Neuroimag. (2020) 306:111178. 10.1016/j.pscychresns.2020.111178 32919869

[31] Malone S Brünken R. Hazard perception, presence, and simulation sickness—a comparison of desktop and head-mounted display for driving simulation. Front Psychol. (2021) 12:647723. 10.3389/fpsyg.2021.647723 33967907 PMC8100057

[32] Hampsey E Meszaros M Skirrow C Strawbridge R Taylor RH Chok L. Protocol for rhapsody: a longitudinal observational study examining the feasibility of speech phenotyping for remote assessment of neurodegenerative and psychiatric disorders. BMJ Open. (2022) 12:e061193. 10.1136/bmjopen-2022-061193 35667724 PMC9171270

[33] König A Mallick E Tröger J Linz N Zeghari R Manera V. Measuring neuropsychiatric symptoms in patients with early cognitive decline using speech analysis. Eur Psychiatry J Assoc Eur Psych. (2021) 64:e64. 10.1192/j.eurpsy.2021.2236 34641989 PMC8581700

[34] Mahon E Lachman ME. Voice biomarkers as indicators of cognitive changes in middle and later adulthood. Neurobiol Aging. (2022) 119:22–35. 10.1016/j.neurobiolaging.2022.06.010 35964541 PMC9487188

[35] Klasnja P Hekler EB Shiffman S Boruvka A Almirall D Tewari A. Microrandomized trials: an experimental design for developing just-in-time adaptive interventions. Health Psychol Off J Div Health Psychol Am Psychol Assoc. (2015) 34S:1220–8. 10.1037/hea0000305 26651463 PMC4732571

[36] Kramer J-N Künzler F Mishra V Presset B Kotz D Smith S. Investigating intervention components and exploring states of receptivity for a smartphone app to promote physical activity: protocol of a microrandomized trial. JMIR Res Protocols. (2019) 8:e11540. 10.2196/11540 30702430 PMC6374735

[37] Nahum-Shani I Smith SN Spring BJ Collins LM Witkiewitz K Tewari A. Just-in-time adaptive interventions (JITAIs) in mobile health: key components and design principles for ongoing health behavior support. Ann Behav Med. (2018) 52:446–62. 10.1007/s12160-016-9830-8 27663578 PMC5364076

[38] Schembre SM Liao Y Robertson MC Dunton GF Kerr J Haffey ME. Just-in-time feedback in diet and physical activity interventions: systematic review and practical design framework. J Med Internet Res. (2018) 20:e8701. 10.2196/jmir.8701 29567638 PMC5887039

[39] Golbus JR Dempsey W Jackson EA Nallamothu BK Klasnja P. Microrandomized trial design for evaluating just-in-time adaptive interventions through mobile health technologies for cardiovascular disease. Circul Cardiovasc Qual Outcomes. (2021) 14:e006760. 10.1161/CIRCOUTCOMES.120.006760 33430608 PMC8887814

[40] Goldstein SP Zhang F Klasnja P Hoover A Wing RR Thomas JG. Optimizing a just-in-time adaptive intervention to improve dietary adherence in behavioral obesity treatment: protocol for a microrandomized trial. JMIR Res Protocols. (2021) 10:e33568. 10.2196/33568 34874892 PMC8691411

[41] Karyotaki E Efthimiou O Miguel C Bermpohl FM Furukawa TA Cuijpers P. Internet-based cognitive behavioral therapy for depression: a systematic review and individual patient data network meta-analysis. JAMA Psychiatry. (2021) 78:361–71. 10.1001/jamapsychiatry.2020.4364 33471111 PMC8027916

[42] Teepe GW Fonseca AD Kleim B Jacobson NC Sanabria AS Car LT. Just-in-time adaptive mechanisms of popular mobile apps for individuals with depression: systematic app search and literature review. J Med Internet Res. (2021) 23:e29412. 10.2196/29412 34309569 PMC8512178

[43] Keller R Hartmann S Teepe GW Lohse K-M Alattas A Car LT. Digital behavior change interventions for the prevention and management of type 2 diabetes: systematic market analysis. J Med Internet Res. (2022) 24:e33348. 10.2196/33348 34994693 PMC8783286

[44] Spruijt-Metz D Marlin BM Pavel M Rivera DE Hekler E De La Torre S. Advancing behavioral intervention and theory development for mobile health: the HeartSteps II protocol. Int J Environ Res Public Health. (2022) 19:2267. 10.3390/ijerph19042267 35206455 PMC8872509

